# Local and Systemic Immunologic Profiles Differentiate Accepted and Rejected Islet Grafts in a Rat Anterior Chamber Model

**DOI:** 10.1155/jimr/2847589

**Published:** 2026-06-21

**Authors:** Jae-young Lee, Choun-Ki Joo, Asif I. Shawl, Hyun Soo Lee

**Affiliations:** ^1^ Department of Ophthalmology, Eunpyeong St. Mary’s Hospital, College of Medicine, The Catholic University of Korea, Seoul, 03312, Republic of Korea, catholic.ac.kr; ^2^ Saint Mary’s Eye Center, Seoul, 06531, Republic of Korea; ^3^ Catholic Institutes of Visual Science, Catholic University of Korea College of Medicine, Seoul, Republic of Korea, catholic.ac.kr; ^4^ Department of Molecular Medicine, The Scripps Research Institute, 10550, North Torrey Pines Road, La Jolla, 92037, California, USA, scripps.edu

## Abstract

The anterior chamber of the eye (ACE) provides a minimally invasive, immune‐privileged site for pancreatic islet engraftment. However, loss of immune privilege during revascularization can trigger inflammation and graft rejection. In this study, we compared ocular and systemic immunologic responses associated with accepted and rejected islet allografts in the ACE. Lewis rat recipients underwent ACE allogeneic islet transplantation without immunosuppressive agents. Ocular grafts and spleens were examined by histology and immunofluorescence for effector and regulatory T‐cell (Treg) populations. Systemic and local cytokines were also quantified and compared between accepted and rejected groups. Graft function (glycemic control) was monitored for 30 days, and diabetic retinopathy development was evaluated histologically. We found glycemic control was lost by 3 weeks in allo‐rejected rats, whereas allo‐accepted rats maintained euglycemia throughout this period. Rats with accepted grafts demonstrated reduced local immune cell infiltration in ACE locally and increased splenic Foxp3^+^ Treg systemically. In contrast, rats with rejected grafts exhibited extensive T‐cell infiltration in both the eye and spleen, decreased splenic Treg populations, elevated local and systemic inflammatory cytokines, and worsened glucose control and diabetic retinal development. Taken together, these results suggest that integrated profiling of local and systemic immunologic signatures effectively distinguishes accepted from rejected ACE islet allografts, and early postoperative monitoring of interleukin 6 (IL‐6), interferon gamma (IFN‐γ), and IL‐10 within the first 3 weeks could facilitate timely, targeted immunosuppression therapy.

## 1. Introduction

Pancreatic islet transplantation is a cornerstone therapy for type 1 diabetes, improving glycemic regulation, reducing exogenous insulin requirements, and enhancing patients’ quality of life by mitigating hypoglycemia‐related complications [1, 2]. Transplantation into the anterior chamber of the eye (ACE) offers an immune‐privileged microenvironment and high oxygen tension that favor islet engraftment [3, 4]. These advantages have advanced the ACE as a promising site, including phase I clinical trials [4]. Nonetheless, islet loss commonly occurs 14–21 days after transplantation due to T‐cell infiltration and inflammation, leading to immune rejection and the need for immunosuppressive therapy [5, 6].

Immunosuppression is essential to suppress T‐cell‐mediated immune responses that drive graft failure, yet many of these agents may compromise islet viability and induce systemic complications [7]. Consequently, strategies to minimize or avoid systemic immunosuppression are a priority, and localized immunomodulation has emerged as a viable alternative [6, 8]. Currently, selective immunomodulation with polyclonal antibodies or interleukin 2 (IL‐2) receptor antagonists remains standard for initial allotransplantation to limit systemic toxicity [9]. The ACE further reduces rejection through anterior chamber–associated immune deviation and provides unmatched access through the transparent cornea [10, 11]. To optimize the dosing and timing of immunosuppressive agents, biomarkers capable of predicting and differentiating rejection‐related immune responses after transplantation are needed to limit standard immunosuppression.

The biomarkers to screen, predict, and distinguish posttransplant immune responses could enable tailored immunosuppression and improve long‐term outcomes [12]. Serum cytokine profiling captures host immune dynamics, with prior studies linking IL‐10, interferon gamma (IFN‐γ), and IL‐6 to islet graft dysfunction and transplant outcomes [13–15]. IL‐6 promotes T‐cell activation and proliferation, creating an inflammatory milieu that damages the graft and impairs regulatory T‐cell (Treg) function, thereby contributing to allograft rejection [16, 17]. In contrast, IFN‐γ drives T‐cell infiltration into allografts by inducing cytokine production and mediating acute rejection mechanisms, whereas IL‐10 suppresses the expression of MHC (major histocompatibility complex) class II molecules and attenuates antigen presentation, central to dampening allogeneic responses [18, 19]. Such immune profiles may enable the stratification of transplant recipients based on their risk of rejection and tolerance, enabling tailored immune modulation and optimized therapeutic strategies.

This study aimed to characterize both local and systemic immunologic alterations underlying transplantation outcomes in a nonimmunosuppressed ACE islet graft model and to determine whether early immune changes in these compartments predict long‐term (10 weeks) graft stability, sustained metabolic efficacy, and development of diabetes‐associated retinal complications. Such integrated immunologic profiling could inform more rational and targeted immunosuppressive therapies, thereby improving overall posttransplant outcomes.

## 2. Materials and Methods

### 2.1. Animals

Male rats aged 6–8 weeks were housed under specific pathogen‐free conditions. Sprague–Dawley rats were purchased from Charles River Orient Laboratory, and Brown Norway rats were imported from Japan from Orient Bio Inc. All animal experiments complied with the Guide for the Care and Use of Laboratory Animals (National Institutes of Health Publication Number 85–23, revised 1996) and were approved by the Institutional Animal Care and Use Committee of The Catholic University of Korea (IACUC approval No. 2018‐004‐04) [20].

### 2.2. Islet Isolation

Pancreatic islets were isolated from Sprague–Dawley rats using a collagenase method [21]. Briefly, animals were euthanized by cervical dislocation after anesthesia with ketamine (120 mg/kg) and xylazine (20 mg/kg). A V‐shaped incision was made starting at the genital area. The pancreas was distended by infusing Hanks’ balanced salt solution (HBSS) containing 0.15 mg/mL type P collagenase through the bile duct, followed by incubation for 15–20 min at 37°C in a water bath. Islets were isolated, washed with HBSS containing HEPES (4‐(2‐hydroxyethyl)‐1‐piperazineethane‐sulfonic acid) and bovine serum albumin (BSA), and the pellet was resuspended in HBSS. Islets were handpicked to remove residual exocrine tissue and stabilized by overnight culture at 37°C in a humidified incubator (95% air, 5% CO_2_) in RPMI (Roswell Park Memorial Institute) medium supplemented with 10% (v/v) fetal bovine serum and 1% penicillin–streptomycin.

### 2.3. Measurement of [Ca^2+^]i

Dispersed β‐cells, obtained by triturating islets with a pipette, were plated on confocal dishes and cultured in a medium. β‐Cells were identified by their large diameter and granular appearance. Adherent cells were washed with HBSS containing HEPES and 0.1% BSA and loaded with 1 μM Fluo‐4 AM (Molecular Probe, Eugene, OR) at 37°C for 20 min. After washing, cells were incubated with glucose supplemented with a hybrid compound with potent insulin‐releasing properties. Changes in [Ca^2+^]i were recorded at 488/530 nm excitation/emission using an air‐cooled argon laser system [22]. The emission at 530 nm was collected with a photomultiplier. Images were acquired every 9 s using a confocal microscope (Nikon, Japan). [Ca^2+^]i was calculated using an equation given by Tsien et al. [23], that is, [Ca^2+^]i = *K*_*d*_ (*F* − *F*_min_)/(*F*_max_ − *F*), where *K*_*d*_ is 450 nM for Fluo‐3 and *F* is the observed fluorescence intensity.

### 2.4. Islets Transplantation

Islet transplantation into the rat ACE was performed as described [24]. Approximately 120 isolated pancreatic islets were aspirated into a blunt 26‐gauge cannula. In anesthetized recipients (Sprague–Dawley or Brown Norway), a horizontal corneal puncture was created with a 26‐gauge needle; the cannula was inserted through the opening, and the islets were gently injected onto the iris under microscopic guidance. All recipients received prophylactic antibiotics but no immunosuppressive agents.

Blood glucose in transplanted rats was measured three times per week using a glucose analyzer (Accu‐Chek). To establish the diabetic model, rats with pretransplant blood glucose levels ≥250 mg/dL were selected for islet transplantation. The allo‐rejected group was defined as recipients who failed to maintain blood glucose below 250 mg/dL during the 30‐day follow‐up period and exhibited a histologic loss of islets. Animals with iris hemorrhage due to procedural complications or without postgraft glycemic control were excluded.

For the glucose tolerance test at 30 days, rats were fasted for 6 h, administered glucose (1 g/kg, intraperitoneally), and blood samples were collected at 0–120 min. Blood glucose was measured by the glucose oxidase method using a glucose analyzer (Accu‐Chek).

### 2.5. Hematoxylin and Eosin Staining

**Figure 1:**
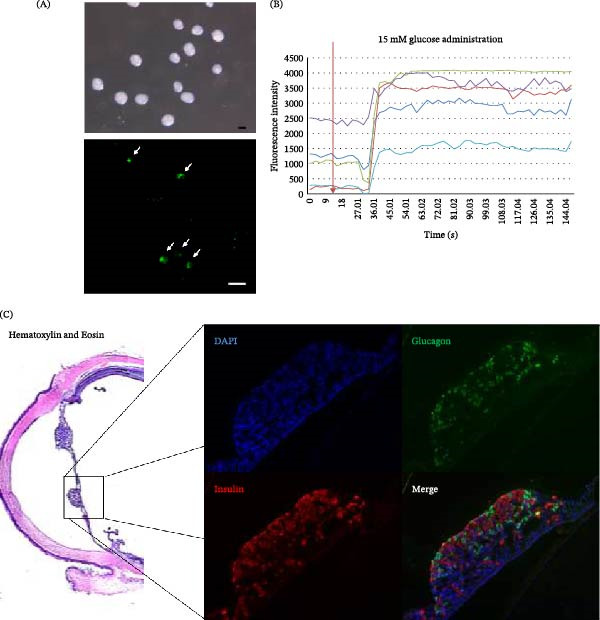
In vitro evaluation of Ca^2+^ signaling in pancreatic β‐cells from the isolated pancreas and validation of the pancreatic transplantation model. (A) Representative surgical microscope images (Zeiss; 10×; scale bar, 100 µm) of pancreatic islets immediately after isolation and confocal micrographs (100×; scale bar, 100 µm) of islets stained with Fluo‐4 AM (arrows). (B) Representative Ca^2+^ activation traces in isolated islet cells loaded with Fluo‐4 AM after high glucose (15 mM) stimulation. (C) Identification and validation of morphological engraftment of transplanted pancreatic cells using hematoxylin and eosin (H&E) staining and fluorescence staining for insulin (red) and glucagon (green) in an anterior pancreas transplantation model. The figures present representative results from three independent experiments.

Cryosectioned tissues were stained with hematoxylin and eosin. Images were acquired on a DMI 5000B microscope (Leica, Wetzlar, Germany) of the grafted islets (100×) and spleen (200×). Stitched images illustrate post graft islet morphology (Figure 1C).

### 2.6. Immunolabeled Tissue Staining

Tissues (eyes and spleens) were fixed in 4% paraformaldehyde and embedded in Tissue‐Tek O.C.T. compound (Cat# 4583, Sakura Finetek, Torrance, CA, USA), sectioned, permeabilized (0.1% Triton X‐100 in phosphate‐buffered saline), and blocked with 5% BSA (Sigma–Aldrich). Sections were incubated overnight at 4°C with primary antibodies (1:200): CD4 (Cat# ab237722, Abcam, Cambridge, MA, USA), CD8 (Cat# ab237709, Abcam), CD25 (Cat# MA1‐90766, Invitrogen), Foxp3 (Cat# 14‐5773‐8, Invitrogen), and VEGF (vascular endothelial growth factor; Cat# sc‐152, Santa Cruz, CA, USA). After washing, sections were incubated at 37°C with secondary antibodies (1:400, Abcam): Alexa Fluor 488 goat anti‐rabbit (ab150077), Alexa Fluor 488 goat anti‐rat (Cat# ab300670), and Alexa Fluor 594 goat anti‐mouse (Cat# ab300670). Apoptosis was assessed using the In Situ Cell Death Detection Kit (Roche Diagnostics, Indianapolis, IN, USA) according to the manufacturer’s instructions. Sections were mounted with DAPI (4′,6‐diamidino‐2‐phenylindole)–containing medium (Vector Laboratories, Burlingame, CA, USA) and imaged on an Axiovert 200 fluorescence microscope at ×200 (Carl Zeiss, Oberkochen, Germany). Immunofluorescence images were quantified using ImageJ by measuring the proportion of positively stained areas or counting positively stained cells. Each experimental group comprised five rats, with masked analysis of the mean value from three representative sections (100× fields) per animal.

### 2.7. Enzyme‐Linked Immunosorbent Assay for Cytokine Expression

On days 3, 14, and 28 posttransplant, serum was collected from the tail vein of rats and assayed for rat IFN‐γ (Cat# 439007, BioLegend, San Diego, CA, USA), IL‐17 (Cat# 437907, BioLegend), IL‐6 (Cat# 437107, BioLegend), and IL‐10 (Cat# BMS629, Invitrogen) concentrations. ELISAs (enzyme‐linked immunosorbent assays) were performed according to the manufacturers’ instructions and read at 450 nm using a microplate reader (Molecular Devices, San Jose, CA, USA).

### 2.8. Statistical Analysis

Analyses were performed using Prism Version 5 (GraphPad Software, La Jolla, CA, USA). Data are presented as mean ± standard deviation. Between‐group differences were assessed with the Student’s *t*‐test or 2‐way ANOVA (analysis of variance) with Bonferroni post hoc testing. Kaplan–Meier survival curves were compared using the log‐rank test. *p* < 0.05 was considered statistically significant.

## 3. Results

### 3.1. Glucose‐Induced Ca^2+^ Signaling in Pancreatic Islets and Validation of the ACE Graft Model

Previous studies have shown that glucose elevates intracellular Ca^2+^ in primary pancreatic islets via metabolic coupling [25, 26]. Fluo‐4 AM imaging confirmed the Ca^2+^ elevation in isolated islets (Figure 1A). To evaluate metabolic responsiveness, β‐cells were exposed to high glucose, which elicited sustained Ca^2+^ signals; fluorescence intensity increased accordingly (Figure 1B), supporting the suitability of the islets for transplantation. Engraftment of islets is critical for survival in the ACE model. Engraftment was demonstrated by intraocular double staining for insulin and glucagon. H&E staining showed successful attachment of islets onto the iris after ACE transplantation, and immunofluorescence confirmed their islet identity (Figure 1C).

### 3.2. Allogeneic Islet Loss and Graft Rejection After ACE Transplantation

**Figure 2:**
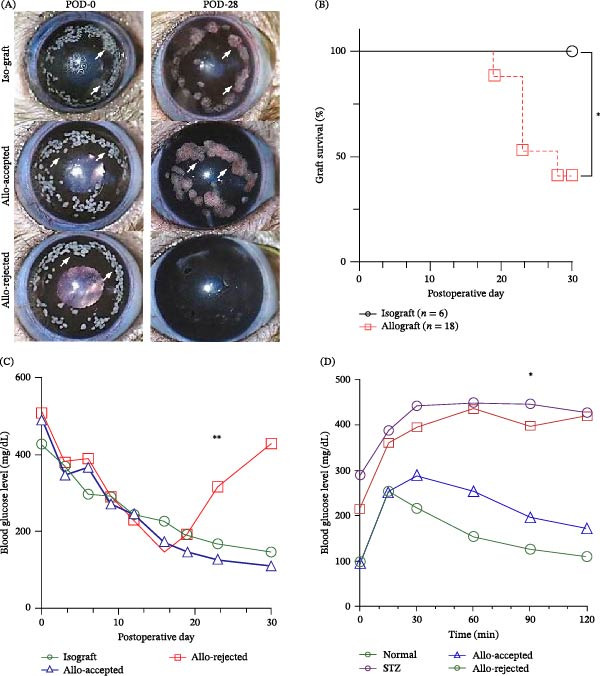
Engraftment, survival, and metabolic parameters after pancreatic islet transplantation. (A) Surgical microscope images at 4 weeks posttransplantation (arrows). (B) Kaplan–Meier survival analysis during 30 days comparing isograft (*n* = 6) and allograft (*n* = 18) recipients; a statistically significant difference was observed by posttransplant day 28, and the groups were compared using the log‐rank test (B;  ^∗^ *p* < 0.05). (C) Blood glucose concentrations tracked for 30 days posttransplantation revealed significantly elevated levels in allograft‐rejected rats (*n* = 10) compared to allograft‐accepted rats (*n* = 8) at day 20 (C;  ^∗∗^ *p* < 0.01). (D) Intraperitoneal glucose tolerance test results showed significantly improved glucose tolerance in the allograft‐accepted group (*n* = 6) relative to the allograft‐rejected group (*n* = 6) ( ^∗^ *p* < 0.05), with no significant difference observed between the allograft‐accepted rats and untreated normal controls. Statistical comparisons among multiple groups were performed using two‐way ANOVA with post hoc testing. Data are presented as the mean ± standard deviation of three independent experiments.

Isolated pancreatic islets were transplanted into the ACE of streptozotocin‐induced diabetic rats. Graft rejection was characterized by progressive loss of intraocular allogeneic islets and hyperglycemia (>250 mg/dL) throughout 30 days posttransplant follow‐up (Figure 2A; isograft, *n* = 6; allograft, *n* = 18). During this time, the allo‐rejected group exhibited a significantly lower graft survival rate, mediated by immune‐mediated rejection (Figure 2B; allo‐rejected, *n* = 10; allo‐accepted, *n* = 8; *p* < 0.05). A significant divergence in blood glucose level between allo‐rejected and allo‐accepted groups became evident at ~20 days posttransplant (Figure 2C; allo‐rejected, *n* = 10; allo‐accepted, *n* = 8; *p* < 0.01). The glycemic regulatory function of the transplanted islets was evaluated at 30 days posttransplantation using intraperitoneal glucose tolerance testing (IPGTT) and revealing a significant difference in glucose homeostasis between groups (Figure 2D; allo‐rejected, *n* = 6; allo‐accepted, *n* = 6; *p* < 0.05). The significance of each survival point was compared using the log‐rank test for the Kaplan–Meier survival curves for each group. No ocular complications, including corneal opacity, conjunctival hyperemia, AC inflammation, abnormal lid blinking, or intraocular pressure‐related corneal edema, were observed in either group.

### 3.3. Local T‐Cell Infiltration and Islet Injury in Accepted Versus Rejected Grafts

**Figure 3:**
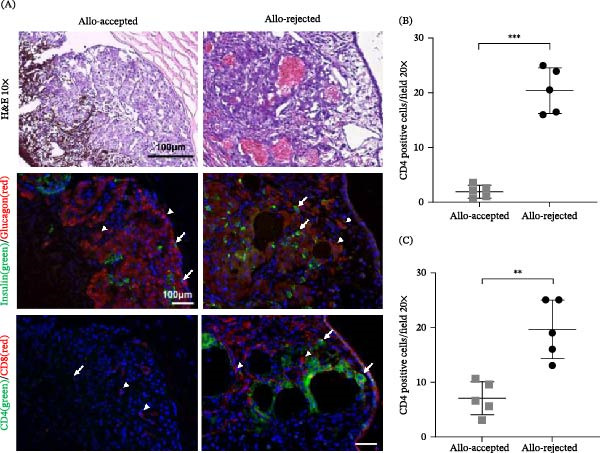
Hematoxylin and eosin (H&E) and immunofluorescence staining of anterior segment of eye cross‐sections to evaluate immune cell infiltration. (A) Representative H&E‐stained sections of the eye. The whole eyeball was sectioned and immunofluorescence stained for insulin (arrows) and glucagon (arrowheads). Mobilized T‐cells were detected by CD4 (arrows) and CD8 (arrowheads) staining (magnification, 200×; scale bar, 100 µm). (B) Quantitative analysis revealed a significantly higher number of CD4^+^ cells in the allo‐rejected group compared to the allo‐accepted group ( ^∗∗∗^ *p* < 0.001). (C) CD8^+^ cell counts were significantly increased in the allo‐rejected group compared to the allo‐accepted group ( ^∗∗^ *p* < 0.01). Data represent mean ± standard deviation of three independent experiments, *n* = 5 rats per group, from independent randomized animals per group, and analyzed in a blinded manner, with multiple sections per eye analyzed for the average value. Statistical differences between groups were assessed using Student’s *t*‐test.

**Figure 4:**
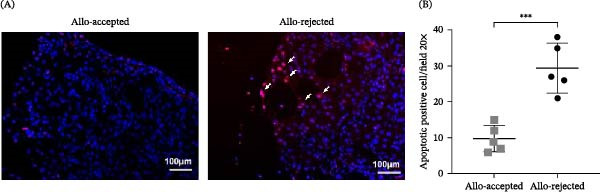
Assessment of cell death in grafts 30 days posttransplantation. (A) Representative images from the TUNEL assay (apoptotic cells, red (arrow); DAPI counterstain, blue); magnification, 200×; scale bar, 100 µm. (B) Quantification of TUNEL‐positive cells. The allo‐rejected group showed significantly higher rate of apoptosis compared to the allo‐accepted group (B,  ^∗∗∗^ *p* < 0.001). Data represent mean ± standard deviation of three independent experiments, *n* = 5 rats per group, from independent randomized animals per group, and analyzed in a blinded manner, with multiple sections per eye analyzed for the average value. Between‐group differences were assessed using Student’s *t*‐test.

Islet loss due to rejection impairs insulin production [27]. In rejected grafts, immunofluorescence staining revealed an overlap of insulin and glucagon expression, indicating disrupted cellular organization, whereas accepted grafts exhibited distinct segregation of these markers (Figure 3A). CD4 and CD8 immunostaining showed significantly fewer infiltrating CD4^+^ T‐cells and CD8^+^ T‐cells in allo‐accepted compared to allo‐rejected islets (Figure 3B, C;  ^∗∗∗^ *p* < 0.001 and  ^∗∗^ *p* < 0.01, respectively). T‐cells are known as key mediators of graft injury during rejection [6]. The allo‐rejected group exhibited a higher number of apoptotic cells within injured regions than the allo‐accepted group (Figure 4A, B;  ^∗∗∗^ *p* < 0.001). The significance of each marker was compared between allo‐accepted (*n* = 5) and rejected (*n* = 5) recipients. Between‐group differences were assessed using the Student’s *t*‐test. These results implicate T‐cell infiltration in the pathogenesis of islet loss and subsequent impairment of insulin secretion.

### 3.4. Graft Rejection Recruits Mature Immune Cells From the Spleen

**Figure 5:**
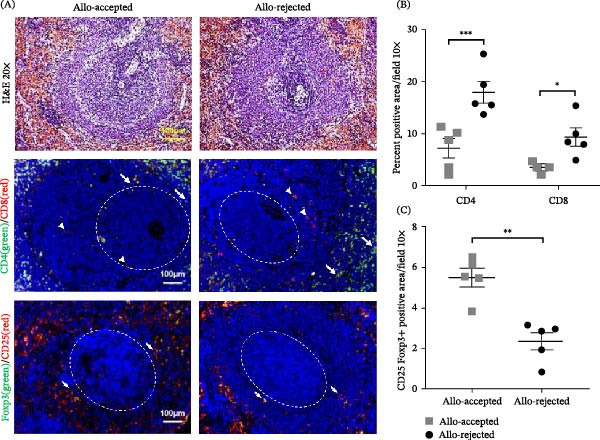
Differentiation and maturation of splenic T‐cell subsets following graft rejection. (A) Representative H&E‐stained spleen sections with immunostaining for CD4 (arrows), CD8 (arrowheads), CD25, and Foxp3 (arrow) to identify immune cell populations in each group (white dotted line: B‐cell follicle area, magnification, 200×; scale bar, 100 µm). (B) Quantification of splenic CD4^+^ and CD8^+^ T‐cells. The allo‐rejected group showed significantly greater expansion of splenic CD4^+^ and CD8^+^ T‐cells in the allo‐rejected group compared to the allo‐accepted group (CD4^+^,  ^∗∗∗^ *p* < 0.001; CD8^+^,  ^∗^ *p* < 0.05). (C) Quantification of CD25^+^ Foxp3^+^ cells demonstrated a significantly larger population in the allo‐accepted group than in the allo‐rejected group (C,  ^∗∗^ *p* < 0.01). Data represent mean ± standard deviation of three independent experiments, *n* = 5 rats per group, from independent randomized animals per group, and analyzed in a blinded manner. For each animal, three to four 100× fields including B‐cell follicles and surrounding T‐cell zones per animal were quantified and averaged to obtain a mean value per animal. Between‐group differences were analyzed using two‐way ANOVA with post hoc testing.

Splenic immune cells are major contributors to ocular immune‐mediated graft rejection. Spleen morphology was evaluated with H&E staining, and immunofluorescence quantification of CD4^+^ ( ^∗∗∗^ *p* < 0.001), CD8^+^ ( ^∗^ *p* < 0.05), and Treg markers was performed to assess positive areas at 30 days (Figure 5; *n* = 5 per group). In the representative immunofluorescence images, the B‐cell follicle areas within the spleen are indicated by white dotted lines, and CD25^+^Foxp3^+^ cells were observed predominantly in the surrounding T‐cell zones. The significance of each CD4^+^CD8^+^ and CD25^+^Foxp3^+^ marker was compared using a two‐way ANOVA multiple comparisons test in the allo‐accepted group and the allo‐rejected group. The allo‐accepted group showed significantly reduced CD4^+^ and CD8^+^ positive areas compared to the allo‐rejected group (Figure 5B). By contrast, the allo‐accepted group showed an increased area of CD25^+^ Foxp3^+^ Tregs relative to the allo‐rejected group (Figure 5C; *n* = 5 per group;  ^∗∗^ *p* < 0.01).

### 3.5. Temporal Dynamics of Systemic Cytokine Levels During Allograft Rejection

**Figure 6:**
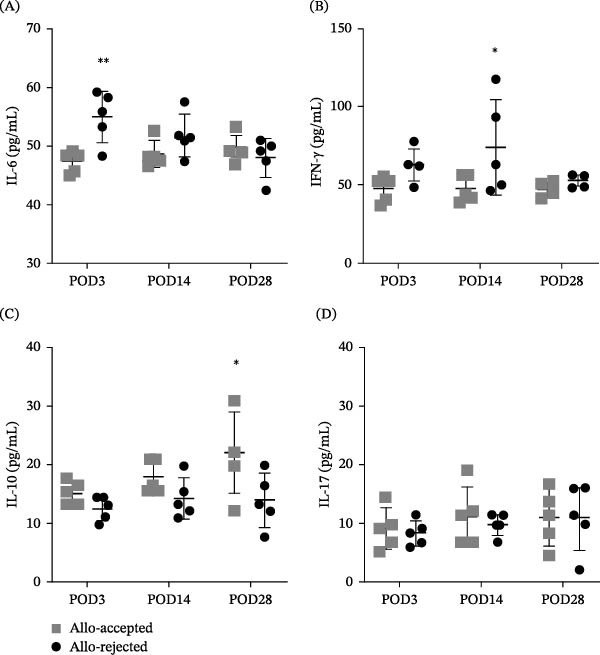
Serum concentrations of proinflammatory and immunoregulatory cytokines quantified by enzyme‐linked immunosorbent assay. Serum cytokines were measured at days 3, 14, and 28 posttransplantations, capturing immune responses beyond the acute rejection phase. Proinflammatory cytokines included IL‐6, IFN‐γ, and IL‐17; the immunoregulatory cytokine was IL‐10. (A) IL‐6 concentrations were significantly higher in the allo‐rejected group at day 3 posttransplantation ( ^∗∗^ *p* < 0.01). (B) IFN‐γ was significantly increased at 14 days in the allo‐rejected group ( ^∗^ *p* < 0.05). (C) IL‐10 was significantly elevated in the allo‐accepted group at day 28 posttransplantation ( ^∗^ *p* < 0.05). (D) IL‐17 levels showed no significant differences between groups at any time point. Data are expressed as mean ± standard deviation, *n* = 5 rats per group, and are representative of three independent experiments. Between‐group differences were assessed using two‐way ANOVA with post hoc testing.

We longitudinally measured serum cytokines associated with allograft rejection from 3 days up to 4 weeks postislet transplantation. In allo‐rejected rats, IL‐6 increased significantly by day 3 posttransplant (Figure 6A; *n* = 5 per group; *p* < 0.01). T helper 1‐associated IFN‐γ also rose significantly by day 14 in the allo‐rejected group (Figure 6B; *n* = 5 per group; *p* < 0.05). Conversely, the allo‐accepted group exhibited a significant rise in the immunoregulatory cytokine IL‐10 by day 28 (Figure 6C; *n* = 5 per group; *p* < 0.05). No significant differences were observed in IL‐17 levels between groups (Figure 6D; *n* = 5 per group). Cytokine concentrations were measured in duplicate for each sample, and the mean value of technical replicates was used for analysis using two‐way ANOVA with Bonferroni post hoc correction. These cytokine profiles reflect divergent local and systemic immune milieus between accepted and rejected grafts.

### 3.6. Anterior Islet Graft Rejection Induces Retinal Neovascularization

**Figure 7:**
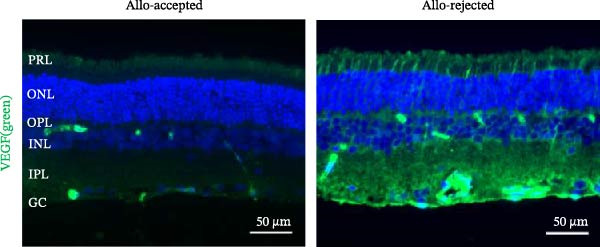
Retinal changes in an STZ‐induced islet transplantation rat model. Retinal neovascularization was evaluated by vascular endothelial growth factor (VEGF) immunostaining at 10 weeks posttransplantation in type 1 diabetes rats. Retinal cross‐sections are shown with VEGF staining in green and DAPI nuclear counterstaining in blue. Retinal layers are labeled as follows: ganglion cell layer (GCL), inner plexiform layer (IPL), inner nuclear layer (INL), outer plexiform layer (OPL), outer nuclear layer (ONL), and photoreceptor layer (PRL). (magnification, 200x; scale bar, 50 µm). Retinas from the allo‐rejected group exhibited marked neovascularization compared to the allo‐accepted group, indicating a pathological angiogenic response associated with graft rejection. The figures present representative data from three independent experiments.

Abnormal neovascularization is a key pathological feature contributing to various ocular immune‐mediated disorders [28, 29]. We assessed retinal diabetic complications with VEGF immunofluorescence during this follow‐up. The allo‐rejected rat retinas showed markedly increased neovascular fluorescence compared to allo‐accepted retinas (Figure 7A). Vascular proliferation was observed within the photoreceptor, inner plexiform, and ganglion cell layers.

## 4. Discussion

A few preclinical studies, as well as an early phase I/II clinical trial in legally blind patients with type 1 diabetes, have demonstrated that ACE islet transplantation is a generally safe and feasible approach without apparent ocular structural complications [30, 31]. However, without adequate immunoregulation, ACE graft islets undergo revascularization, lose immune privilege, initiate the inflammatory responses, and subsequently are eliminated [5, 6]. In this study, early islet loss within 4 weeks posttransplantation was significantly associated with long‐term (10‐week) graft survival and the development of diabetic retinopathy, and early peripheral immune alterations correlated with favorable graft outcomes. Moreover, we identified early posttransplant peripheral blood immune alterations that correlated positively with favorable long‐term graft outcomes. These findings suggest that immune alterations within 3 weeks posttransplantation may serve as prognostic indicators of islet engraftment efficiency and functional viability.

We established the animal model by isolating viable healthy islets, followed by transplantation as autografts into the ACE (Figure 1A, B). Within 1week posttransplant, grafts had engrafted and induced neovascular growth from the iris (Figure 1C). The model was subsequently adapted to an allogeneic context to evaluate graft survival and immune activation. In allografted rats, glucose regulation deteriorated by 3 weeks, concomitant with islet loss (Figure 2A, C). Consistent with findings by Abdulreda et al. [11], host T‐cell infiltration within the ACE facilitates allograft rejection. Notably, despite the absence of immunosuppression in this study, 44% of grafts survived through the observation period, with surviving grafts sustaining insulin secretion, as assessed by IPGTT at 4 weeks (Figure 2B, D). Subsequent immunological analyses were conducted on animals exhibiting graft loss versus accepted.

Immunofluorescence of graft loss revealed extensive CD4^+^ and CD8^+^ T‐cell infiltration (Figure 3), consistent with prior reports of ACE graft rejection [5, 32]. CD4^+^ and CD8^+^ T‐cells are major effectors of islet destruction. CD8^+^ T‐cells mediate target‐cell apoptosis via perforin–FasL, while CD4^+^ T‐cells amplify inflammation and cytokine release, thereby compromising islet viability and function [33, 34]. In this study, islets with pronounced CD4^+^ or CD8^+^ T‐cell infiltration exhibited marked cellular loss. Even morphologically intact grafts showed increased apoptosis in recipients with early incipient inflammation (Figure 4). The spleen is a key reservoir of immune cells recruited during intraocular inflammation and immune privilege disruption. Alloantigen‐specific T‐cells originating from the spleen migrate to the graft through cytokine‐mediated mechanisms to facilitate islet destruction [10], whereas splenic Foxp3^+^ Tregs can migrate to the ACE and induce anterior chamber–associated immune deviation [35]. In our ACE islet allograft recipients, splenic immunofluorescence in ACE islet allograft recipients demonstrated differential modulation of effector and Treg populations between allo‐rejected and allo‐accepted groups (Figure 5). These findings suggested that, even without immunosuppression agents, distinct immune responses differentiate allo‐accepted from allo‐rejected recipients, and early detection of these immune signatures may influence graft survival deterministically.

We observed a significant increase in IL‐6 in the allo‐rejected group versus the allo‐accepted group on posttransplant day 3 (Figure 6A). IL‐6 is predominantly secreted by dendritic cells upon alloantigen exposure, driving early inflammation and promoting CD8^+^ T‐cell infiltration through the activation of alloreactive CD4^+^ T‐cells [36, 37]. Inhibition of IL‐6 signaling has been associated with decreased oxidative stress via NF‐κB inactivation during graft loss [17, 37]. Prior clinical studies have demonstrated a correlation between elevated serum IL‐6 levels and allograft survival in type 1 diabetes patients, highlighting IL‐6 as a critical initiator of inflammation in the islet graft model [38]. Islets are particularly susceptible to inflammatory cytokines such as IL‐6 and IFN‐γ, and IFN‐γ produced by innate immune cells and effector T‐cells can promote neutrophil activation and graft loss [39, 40]. In our study, allo‐rejected recipients showed a significant increased IFN‐γ at week 1, correlating with impaired insulin regulation and reduced graft survival (Figure 6B). The temporal rise of IFN‐γ implicates its role not only in direct cytokine‐mediated islet damage but also in promoting T‐cell differentiation and proliferation critical for graft fate. Our results suggest that early IL‐6 elevation creates a pro‐inflammatory environment that promotes IFN‐γ production and effector T‐cell expansion, contributing to islet injury and graft loss [17, 40].

The allo‐accepted groups showed significantly elevated IL‐10 than the allo‐rejected group at week 4 (Figure 6C). IL‐10 is a pivotal immunoregulatory cytokine in islet transplantation and has been suggested as a biomarker of anti‐inflammatory and immune‐modulatory states associated with and potentially prognostic of graft survival. Clinical studies have shown that elevated serum IL‐10 in islet transplant recipients correlates with higher rates of insulin independence and favorable outcomes [41, 42]. In addition, preclinical studies have demonstrated that IL‐10 contributes to long‐term graft survival and effective immunoregulation in experimental ACE islet transplantation models [11]. IL‐10 can directly protect transplanted islets by promoting the induction and maintenance of Tregs, downregulating Fas expression, and inhibiting caspase‐3 [43–45]. The elevated IL‐10 likely suppresses pathogenic T‐cell activity and suppresses proinflammatory cytokines, including IFN‐γ, thereby facilitating positive graft outcomes after ACE islet transplantation [46, 47]. To our knowledge, no previous animal study has reported 10‐week islet survival without immunosuppression agents in an ACE allograft model [6]. Our study results support the findings from local and systemic immunologic workups at the ACE transplantation site, demonstrating distinct differences between accepted and rejected grafts. Therefore, the later rise in IL‐10 in allo‐accepted recipients likely reflects a regulatory pathway that supports Foxp3^+^ Tregs, suppresses proinflammatory cytokines, and helps maintain islet graft function [19, 47].

Prior studies reported induction of IL‐17A in xenogeneic transplantation models but no significant change in serum IL‐17 in allogeneic grafts [48]. Likewise, studies testing anti–IL‐23R therapy to inhibit T helper 17 differentiation and IL‐17 production found no significant differences in IL‐17 concentrations between treated and control groups [49]. Consistent with these findings, we detected no significant variation in IL‐17 at any time point after islet transplantation (Figure 6D). These results suggest that while IL‐17–mediated pathways may contribute to graft rejection, IL‐17 alone is insufficiently sensitive as a biomarker for islet graft rejection. We also tried to assess cytokines in the aqueous humor but could not detect meaningful changes because of insufficient sampling volume. Notably, prior cytokine‐profiling studies in rodent aqueous humor have documented significant recipient IFN‐γ elevations at the onset of graft rejection [26]. Although these mechanisms are biologically plausible and consistent with previous reports in transplantation, because this study is observational, additional experiments that modulate the relevant signaling pathways in the ACE model will be needed in the future to confirm causal relationships.

During the 10‐week observation period, no specific ocular complications, including corneal opacity and edema, cataract formation, conjunctival redness, severe intraocular inflammation, or abnormal behaviors related to visual discomfort, were observed in either group. However, the long‐term risks of anterior chamber islet transplantation require further evaluation before clinical trials. Although the anterior chamber exhibits ACAID‐mediated immune privilege, immunosuppression could prevent allogeneic graft rejection and maintain euglycemia, as evidenced by our findings on ocular and systemic alloimmune responses in this study. In this study, alterations in local and systemic immunologic responses following ACE islet transplantation without immunosuppression agents were closely associated with subsequent islet loss. Monitoring of these immunologic workups may inform timely immunomodulatory interventions to enhance islet survival. However, additional studies are necessary to establish long‐term validation of immunologic responses for clinical applications. Future investigations will focus on integrating immunological analyses in the established ACE transplantation model to elucidate chronic immune interactions and identify therapeutic targets to further improve graft survival and metabolic outcomes.

## 5. Conclusions

This study systematically characterized local and systemic immune responses associated with islet allograft acceptance and rejection in the anterior chamber transplantation model. Integrated analyses of immune cell infiltration, splenic immune regulation, and serum cytokine profiles revealed distinct immunologic signatures associated with and may serve as prognostic markers of graft outcome. Elevated expression of IL‐6 and IFN‐γ, accompanied by reduced IL‐10 levels, strongly correlated with graft rejection. These findings suggest that disruption of ocular immune privilege following transplantation is accompanied by enhanced alloimmune responses and subsequent changes in retinal vascular development. This study provides critical insights into the immune mechanisms underlying graft outcomes and establishes a basis for targeted immunomodulatory therapies to enhance transplant survival and function.

## Author Contributions

Performed the experiments: Jae‐young Lee, Hyun Soo Lee, Choun‐Ki Joo, and Asif I. Shawl. Analysis of in vivo data: Jae‐young Lee and Hyun Soo Lee. Drafting the manuscript: Jae‐young Lee. Conception and design of study: Hyun Soo Lee. Revised the manuscript critically for important intellectual content: Hyun Soo Lee.

## Funding

This work was supported by the Basic Science Research Program through the National Research Foundation of Korea (NRF), funded by the Ministry of Education (Grants RS‐2022‐NR073455 and RS‐2025‐24683579), R&D and Ministry of Health and Welfare as Korea Health Technology R&D Project (Grant HI17C2012030018), and the Catholic Medical Center Research Foundation made in the program year of 2024.

## Conflicts of Interest

The authors declare no conflicts of interest.

## Data Availability

The data that support the findings of this study are available from the corresponding author upon reasonable request.
